# Utilization of 2:1 Internal Resonance in Microsystems

**DOI:** 10.3390/mi9090448

**Published:** 2018-09-08

**Authors:** Navid Noori, Atabak Sarrafan, Farid Golnaraghi, Behraad Bahreyni

**Affiliations:** School of Mechatronic Systems Engineering, Simon Fraser University, Surrey, BC V5A 1S6, Canada; nnoori@sfu.ca (N.N.); asarrafa@sfu.ca (A.S.); mfgolnar@sfu.ca (F.G.)

**Keywords:** 2:1 internal resonance, energy transfer, micromachined resonators, nonlinear modal interactions, perturbation method

## Abstract

In this paper, the nonlinear mode coupling at 2:1 internal resonance has been studied both analytically and experimentally. A modified micro T-beam structure is proposed, and the equations of motion are developed using Lagrange’s energy method. A two-variable expansion perturbation method is used to describe the nonlinear behavior of the system. It is shown that in a microresonator with 2:1 internal resonance, the low-frequency mode is autoparametrically excited after the excitation amplitude reaches a certain threshold. The effect of damping on the performance of the system is also investigated.

## 1. Introduction

Microresonators are microfabricated devices that can be operated at their resonance. These microdevices are used in a variety of applications including timing references, filters, sensors, actuators, etc. [1]. Microresonators are typically used within their linear range of operation. Nevertheless, there has been an increasing interest in operating the microresonators at the nonlinear mode of operation to enhance their performance [2,3,4,5]. Unavoidable nonlinearities can be found in many micro or macro systems. They can cause severe impact on performance of high quality-factor (Q) MEMS devices. In some cases, neglecting the presence of nonlinearities can lead to erroneous predictions of system’s dynamic [6]. It is not always the case that engineers avoid the nonlinearities because of the degradation in the system’s performance and the unwanted outcomes. MEMS designers can also beneficially exploit nonlinearities, e.g., mechanical or electrical nonlinearities, in the design of microdevices for various purposes, e.g., sensing, actuation, timing, and signal processing [7].

Nonlinear mode coupling is one of the outcomes of the presence of nonlinearities in the system. Nonlinear mode coupling results in transfer of energy from an intentionally excited mode to other modes of vibration. One of the mechanisms of nonlinear mode coupling is internal resonance. Internal resonance (also known as autoparametric excitation) refers to the transfer of energy from one vibrational mode to another mode where the resonance frequencies of these vibrational modes are commensurable or nearly commensurable. Internal resonance can be used in various applications including mass sensing, inertial sensing [8], energy harvesting [9], and noise suppression [10].

In a system with nonlinear 2:1 internal resonance: (1) there exists a frequency ratio of 2:1 between the natural frequencies of two resonant modes, and (2) quadratic nonlinearities couple the vibrational modes [11]. Due to this nonlinear mode coupling, energy can be channelled from one mode to another mode. There is an interesting nonlinear phenomenon in the systems with 2:1 internal resonance, which is known as saturation. When the system is excited at its primary mode, the amplitude of this mode increases linearly with an increase in the excitation amplitude until the modal amplitude reaches a specific threshold. After this point, the amplitude of the primary mode remains at a constant value and the excessive energy acquired by the increase in the excitation amplitude channels to the secondary mode. One of the simplest examples of a system with 2:1 internal resonance is a spring-pendulum system where energy is being exchanged between spring mode and pendulum mode [12,13,14]. A similar study to investigate the internal resonance in a micro H-shaped microdevice has been done by Sarrafan et al. [15].

In this paper, a microresonator with 2:1 internal resonance is introduced. The mathematical model of the system is developed and solved by using a perturbation method. Reduced-order analysis of the structure in CoventorWare© software (ver. 2012, Coventor, Inc., Cary, NC, USA) is also explained. Finally, fabricated microdevice is characterized, and the experimental results showing nonlinear mode coupling are thoroughly discussed.

## 2. Materials and Methods

**Figure 1:**
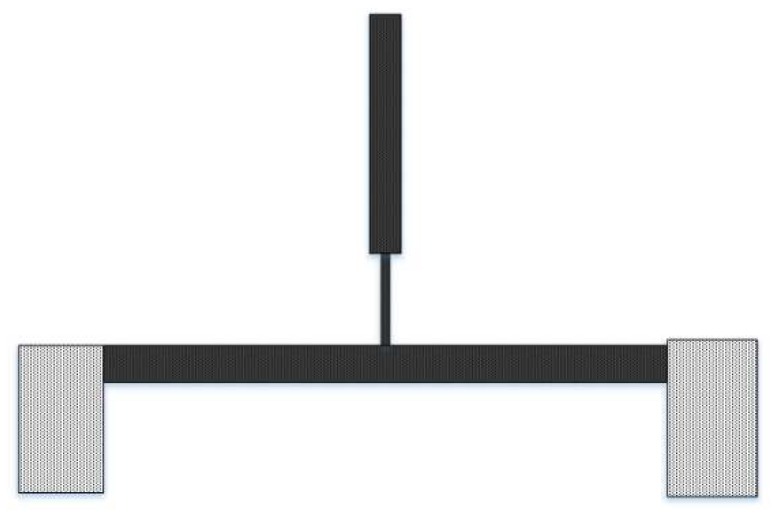
Schematic view of the modified micro T-beam.

A modified micro T-beam structure is designed to operate based on the principle of nonlinear 2:1 internal resonance. The schematic of the microdevice is shown in Figure 1. This design idea originated from the T-beam design in [16]. The modified T-beam structure consists of three beams: (1) the drive beam (bottom beam) which is anchored to the substrate and used for actuating the structure, (2) the narrowed beam which is connected to the center of the drive beam and has a relatively low stiffness, and (3) the sense beam which is connected to the narrowed beam and is used for sensing the response of the system.

**Figure 2:**
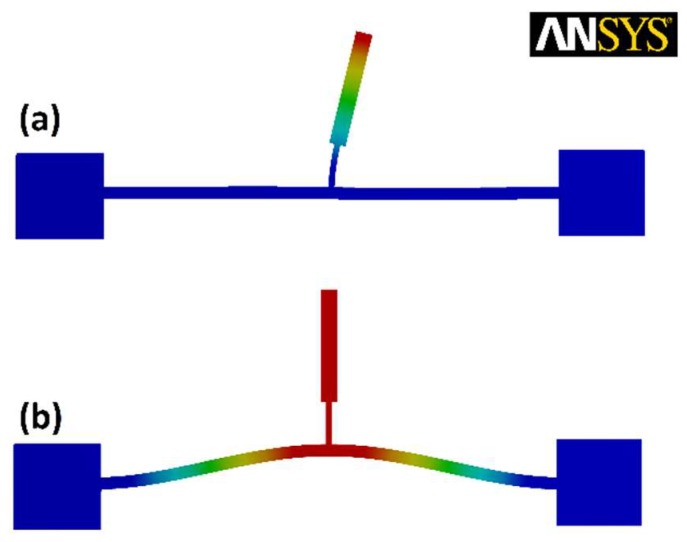
In-plane mode shapes of the micro T-beam structure obtained from ANSYS^®^ FEM simulation: (**a**) the first mode (**b**) the second mode.

Figure 2 shows the desired mode shapes of the structure from finite element method (FEM) simulations in ANSYS^®^ software. The structure is designed to have a frequency ratio of 2:1 between its first and second structural modes. It is expected that exciting the system at its second resonance frequency excites the first vibrational mode autoparametrically.

**Figure 3:**
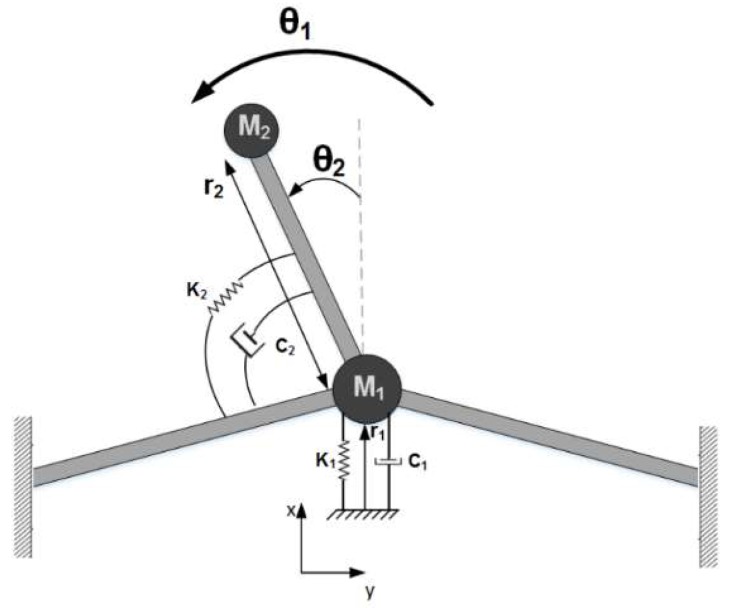
Lumped element representation of micro T-beam structure.

To better understand the nonlinear mode coupling and energy transfer between vibrational modes, the equations of motion of the system can be solved by perturbation method. The first step in this process is to model the system mathematically. The modified T-beam structure can be described by using lumped elements, shown in Figure 3. In this model, *m*_1_ and *m*_2_ represent the effective mass of the drive beam and the sense beam, respectively. Similarly, *c_i_* and *k_i_* are the effective damping coefficient and the spring constant of the beams, respectively. By using Lagrange’s energy method, the equations of motion can be written as:

(1) $$\begin{array}{l} \left(m_{1}+m_{2}){\ddot{r}}_{1}+c_{1}{\dot{r}}_{1}+k_{1}r_{1}-m_{2}r_{2}(({\ddot{\theta }}_{1}\sin(\theta_{2})+{\dot{\theta }}_{1}^{2}\cos(\theta_{2})+2{\dot{\theta }}_{1}{\dot{\theta }}_{2}\cos(\theta_{2})\right)\\ -m_{2}r_{2}({\ddot{\theta }}_{2}sin(\theta_{2})+{\dot{\theta }}_{2}^{2}\cos(\theta_{2}))-(m_{1}+m_{2})r_{1}{\dot{\theta }}_{1}^{2}=F_{drive}(t)\\ m_{2}r_{2}^{2}{\ddot{\theta }}_{2}+c_{2}{\dot{\theta }}_{2}+k_{2}\theta_{2}-m_{2}r_{2}{\ddot{r}}_{1}\sin(\theta_{2})+2m_{2}{\dot{r}}_{1}r_{2}{\dot{\theta }}_{1}\cos(\theta_{2})\\ +m_{2}r_{1}r_{2}({\ddot{\theta }}_{1}\cos(\theta_{2})+{\dot{\theta }}_{1}^{2}\sin(\theta_{2}))=-m_{2}r_{2}^{2}{\ddot{\theta }}_{1} \end{array}$$

These equations will then be non-dimensionalized and scaled. A perturbation method named as two-variable expansion method is used to study the nonlinear behaviour of the system. Due to the lengthy nature of the perturbation method, only the final solution of the system is provided here, with additional details about each step in the perturbation method provided in [5]. The final perturbation solution of the modified T-beam structure for *ρ* and *θ*, the non-dimensionalized amplitudes of the drive beam and the sense beam, respectively, are found from [5]:

(2) $$\begin{array}{l} \rho =\frac{2\omega_{2}}{\omega_{1}^{2}}\sqrt{{\left(\sigma_{1}+\sigma_{2}\right)}^{2}+\mu_{2}^{2}}\cos(\Omega_{1}\tau -\gamma_{1})+O(\varepsilon )\\ \theta =\frac{1}{\omega_{2}}\sqrt{\frac{\Lambda_{1}\pm \sqrt{f_{1}^{2}\omega_{1}^{6}-\Lambda_{2}^{2}}}{m\omega_{1}}}\cos(\frac{1}{2}\Omega_{1}\tau -\frac{\gamma_{1}+\gamma_{2}}{2})+O(\varepsilon ) \end{array}$$

Parameters in Equation (2) are defined in Table 1.

**Figure 4:**
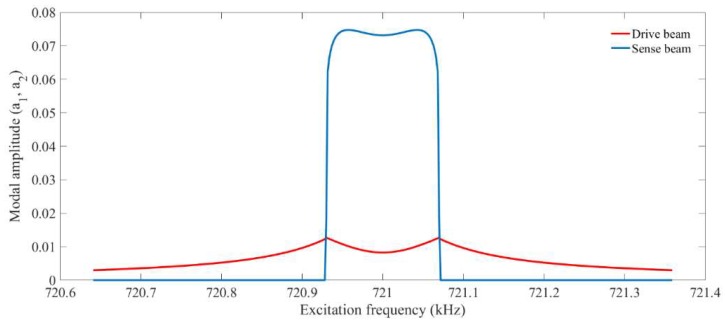
Simulated Nonlinear frequency sweep achieved from two-variable perturbation solution.

**Figure 5:**
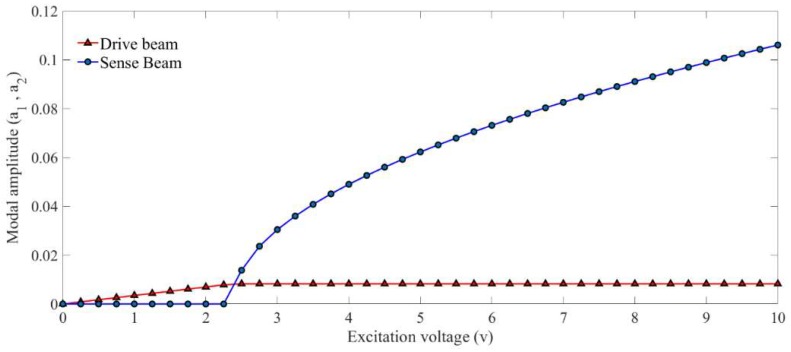
Simulated saturation curve by two-variable perturbation solution.

Figure 4 shows the simulated nonlinear frequency response of the system from perturbation solution. As it can be seen, as the energy starts to transfer between the modes, the amplitude of the drive mode drops and the amplitude in sense mode grows. It can also be seen that a nearly flat region is formed in the frequency response of the sense mode. Figure 5 illustrates the saturation phenomenon. It can be seen that the system behaves linearly before the amplitude of the drive mode reaches a certain point and there is no energy transfer between vibrational modes. However, the system starts to behave nonlinearly as the amplitude in the drive mode reaches the threshold.

**Figure 6:**
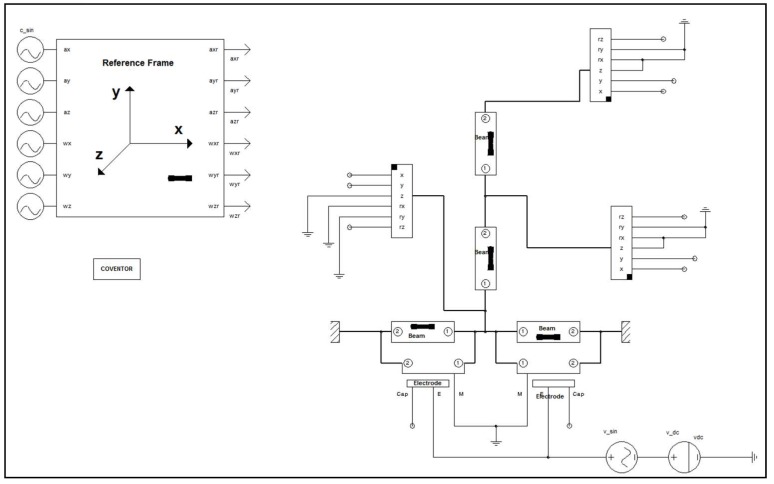
System schematic in CoventorWare© Architect.

Numerical simulation in CoventorWare© to model the dynamical behaviour can be helpful for the proper design of the system. FEM analysis is also an essential step in the design process of the system to ensure that resonance frequencies of the two desired structural modes are close to the 2:1 ratio (*ω*_2_ = 2*ω*_1_). Dimensional adjustments are done based on these results from FEM analysis to ensure the target 2:1 frequency ratio. Architect module in CoventorWare© is being used to perform a reduced-order modelling and simulation of the system. Figure 6 shows the schematic view of the modelled system in the CoventorWare© Architect.

**Figure 7:**
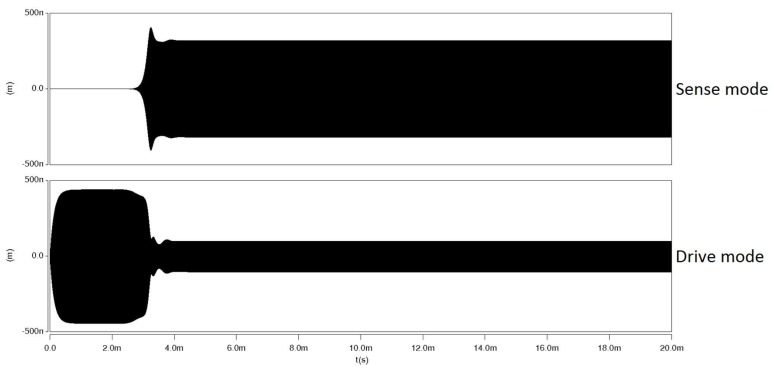
Time-domain response from transient simulation in CoventorWare© showing the transfer of energy between vibrational modes.

The electrostatic excitation is used in the simulations. A 40 V DC voltage is applied to the electrodes, and the drive beam is excited to reach its resonance by applying appropriate AC voltage to the drive electrode. Figure 7 shows the time response of the system with energy transfer between vibrational modes. As it can be seen in the time response-similar to results from perturbation solution, when internal resonance begins to happen, energy starts to exchange between vibrational modes. The drive mode amplitude starts to drop, and the amplitude of the sense mode simultaneously starts to grow exponentially until the system reaches to the steady state. The exponential growth of the sense mode amplitude reveals the absence of damping during the transfer of energy between modes.

**Figure 8:**
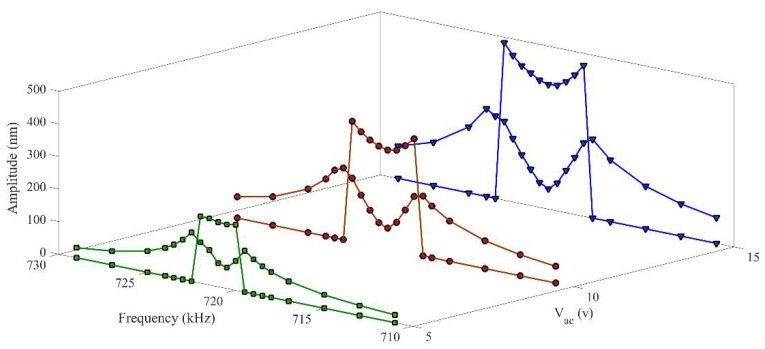
Nonlinear frequency curves in CoventorWare© showing the nonlinear mode coupling between the drive and sense modes.

It is also expected that by increasing the excitation amplitude, the coupling between vibrational modes becomes stronger. Therefore, the higher amplitudes can be observed in both the drive and sense modes. Figure 8 demonstrates the nonlinear frequency response of the system for different excitation amplitudes.

**Figure 9:**
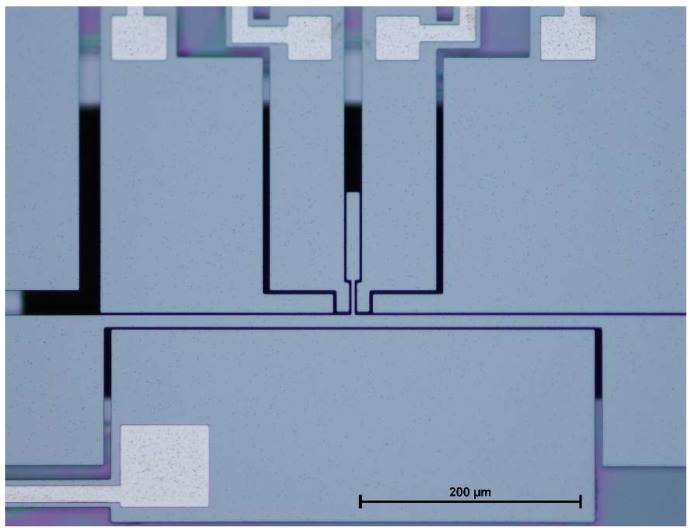
Fabricated device using SOIMUMPS (Silicon-on-Insulator Multi-User MEMS Processes) process.

With the help of the perturbation solution and the CoventorWare© simulations, the modified micro T-beam structure is designed. The microdevice is fabricated by Silicon-on-Insulator Multi-User MEMS Processes (SOIMUMPS). SOIMUMPS is a general purpose microfabrication process introduced by MEMSCAP for micromachining of devices with highly planar surfaces in a SOI framework. This process is a simple 4-mask level SOI patterning and etching. It is a great choice of fabrication for proof of concept purpose. This process has a minimum feature size of 2 µm and the minimum gap between any two silicon parts is also 2 µm. More details about this process can be found in [17]. Figure 9 shows the final fabricated structure.

**Figure 10:**
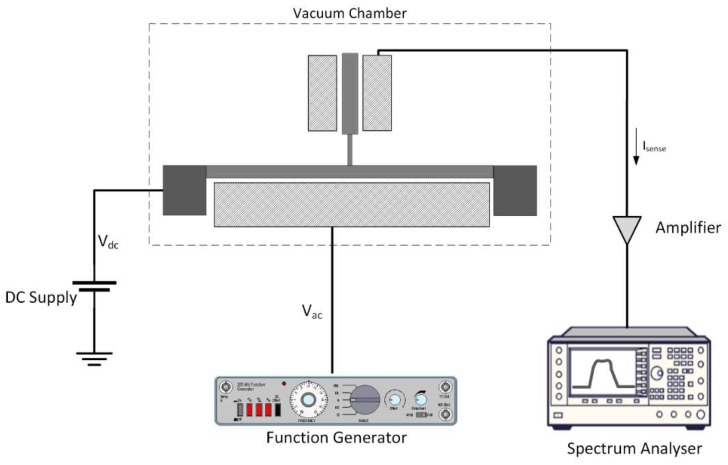
Experimental setup for frequency sweep. Both excitation and sensing are done by electrostatic transduction.

The next step is to perform experimental tests to investigate the nonlinear response of the fabricated structure. Figure 10 depicts the test setup used for the nonlinear frequency sweep test. The experimental setup used to conduct the frequency sweep tests consists of (1) the fabricated modified micro T-beam, (2) vacuum chamber, (3) a DC voltage source, (4) a function generator for excitation, (5) a signal amplifier, (6) spectrum analyzer to monitor the output signal. As it can be seen, the system is being excited to reach its resonance by exciting the drive beam electrostatically. The response of the system is being sensed through the electrostatic electrodes beside the sense beam and is then being amplified before reaching the spectrum analyzer for monitoring.

Resonance frequencies of the fabricated structure are also measured by a network analyzer and specified to be 361.135 kHz (first mode) and 722.590 kHz (second mode). These measurements imply a nearly ideal frequency ratio of 2.001. In the next section, the experimental frequency sweeps and also the effect of damping on the performance of the system are discussed.

## 3. Results and Discussion

**Figure 11:**
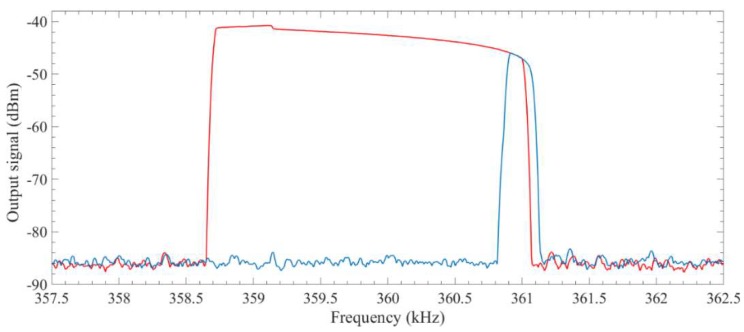
Forward (blue) and backward (red) frequency sweeps showing the nonlinear response of the system.

The drive beam is actuated by an AC signal with a frequency near its resonance frequency in the range of 714 kHz and 724 kHz. Both forward and backward frequency sweeps are acomplished to investigate the nonlinear performance of the structure. Figure 11 shows the sense beam’s response in forward and backward frequency sweeps. It can also be observed in this figure that there is an overlapping region between forward and backward frequency sweeps. This region relates to the frequency range that response of the system is not dependent on the direction of the sweep.

**Figure 12:**
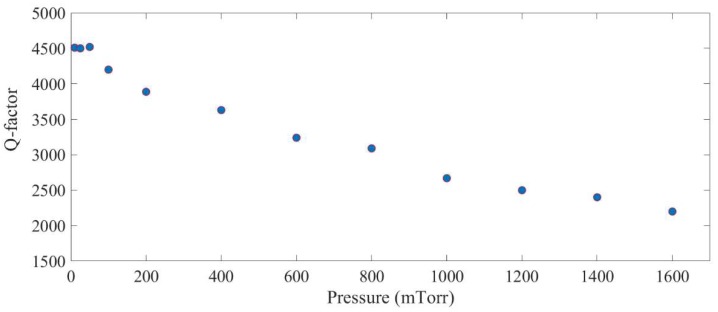
Quality factor of the sense mode of the structure while operating in its linear region.

The next step is to investigate the effect of damping on the nonlinear response of the system. Damping of a resonator can be represented by the quality factor of the system (Q-factor). Quality factor is one of the most important parameters of microresonators which directly relates to the resonance amplitude of the microresonator. In a linear microresonator, a linear relation is expected between Q-factor and amplitude at resonance. Figure 12 shows quality factor of the sense mode of the system within its linear range of operation at different operating pressures. As can be seen, viscous damping dominates the energy loss at pressures above ~100 mTorr. At lower pressures, other sources of energy loss, such as support losses or thermoelastic damping, dominate. As these loss mechanisms are independent of pressure, the quality factor plateaus at pressures less than ~70 mTorr.

**Figure 13:**
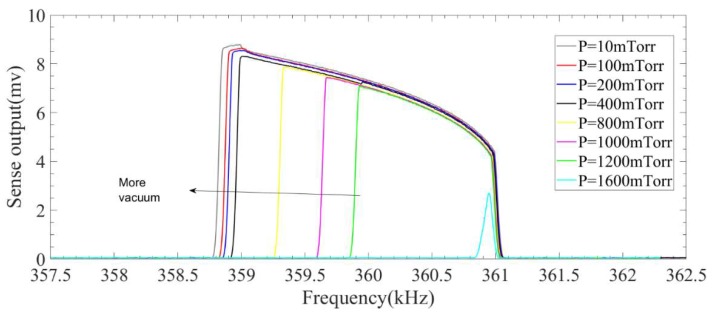
Effect of pressure on the performance of the system in the presence of internal resonance in a backward frequency sweep.

**Figure 14:**
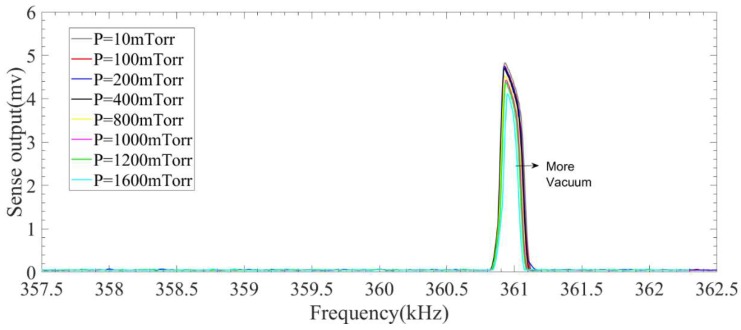
Effect of pressure on the performance of the system in the presence of internal resonance in a forward frequency sweep.

This figure also shows that the quality factor of the system operating in the linear region varies between 4500 and 2200 in the operating pressure of nearly zero (10 mTorr) to 1.6 Torr. A set of experiment is conducted to investigate the effect of operating pressure on the response of the microresonator in the presence of the 2:1 internal resonance. To conduct this test, the vacuum chamber’s pressure is being changed slowly from near vacuum condition (here 10 mTorr) to 1.6 mTorr. After fixing the pressure, frequency sweeps are performed to show the internal resonance phenomenon. Figure 13 and Figure 14 show the nonlinear response of the system in different operating pressures for both backward and forward frequency sweeps.

These figures reveal that unlike the linear case, the amplitude of response in the presence of 2:1 internal resonance does not change significantly for pressures below 1600 mTorr. However, the bandwidth of the response becomes smaller as the operating pressure increases. These results show that microresonators working under internal resonance do not need to be necessarily packaged in near vacuum which can significantly reduce the packaging costs of these MEMS devices.

## 4. Conclusions

A modified micro T-beam with the 2:1 internal resonance was proposed and designed with the help of perturbation solutions and the nonlinear analysis in CoventorWare© Architect. The designed structure was then fabricated by a SOIMUMPS process. Experimental tests on the fabricated structure with a nearly perfect frequency ratio of 2:1 verified the simulation results qualitatively. A significant response enhancement in both forward and backward frequency sweeps were also observed in these results. The response of the system in different operating pressures was studied. It showed that unlike the linear response of the system, response amplitude under the 2:1 internal resonance does not depend significantly on the operating pressure.

## Figures and Tables

**Table 1:** Definition of nondimensionlized parameters for Equation (2).

Nondimensionalized Parameters	Symbol
Drive mode frequency	*ω*_1_
Sense mode frequency	*ω*_2_
Perturbation parameter	*ε*
Detuning frequency ($\Omega_{1}=\omega_{1}+\varepsilon \sigma_{1}$)	*σ*_1_
Detuning frequency ($\omega_{1}=2\omega_{2}+\varepsilon \sigma_{2}$)	*σ*_2_
Drive beam damping	*γ*_1_
Sense beam damping	*γ*_2_
Excitation force amplitude	*f*_1_
Excitation force frequency	Ω_1_
$\omega_{2}\left[4\sigma_{1}(\sigma_{1}+\sigma_{2})-2\mu_{1}\mu_{2}\right]\;$	Λ_1_
$\omega_{2}\left[2\mu_{1}(\sigma_{1}+\sigma_{2})+4\sigma_{1}\mu_{2}\right]$	Λ_2_
